# Efficacy of Plant-Derived Fungicides at Inhibiting *Batrachochytrium salamandrivorans* Growth

**DOI:** 10.3390/jof8101025

**Published:** 2022-09-28

**Authors:** Adrianna Tompros, Mark Q. Wilber, Andy Fenton, Edward Davis Carter, Matthew J. Gray

**Affiliations:** 1 Center for Wildlife Health, Department of Forestry, Wildlife, and Fisheries, University of Tennessee Institute of Agriculture, Knoxville, TN 37996, USA; 2 Institute of Infection, Veterinary and Ecological Sciences, University of Liverpool, Liverpool L69 7ZB, UK

**Keywords:** *Batrachochytrium salamandrivorans*, antifungal, plant-derived, amphibian, salamander, zoospore, minimum inhibitory concentration, minimum fungicidal concentration

## Abstract

The emerging fungal amphibian pathogen, *Batrachochytrium salamandrivorans* (*Bsal*), is currently spreading across Europe and given its estimated invasion potential, has the capacity to decimate salamander populations worldwide. Fungicides are a promising in situ management strategy for *Bsal* due to their ability to treat the environment and infected individuals. However, antifungal drugs or pesticides could adversely affect the environment and non-target hosts, thus identifying safe, effective candidate fungicides for in situ treatment is needed. Here, we estimated the inhibitory fungicidal efficacy of five plant-derived fungicides (thymol, curcumin, allicin, 6-gingerol, and Pond Pimafix^®^) and one chemical fungicide (Virkon^®^ Aquatic) against *Bsal* zoospores in vitro. We used a broth microdilution method in 48-well plates to test the efficacy of six concentrations per fungicide on *Bsal* zoospore viability. Following plate incubation, we performed cell viability assays and agar plate growth trials to estimate the minimum inhibitory concentration (MIC) and minimum fungicidal concentration (MFC) of each fungicide. All six fungicides exhibited inhibitory and fungicidal effects against *Bsal* growth, with estimated MIC concentrations ranging from 60 to 0.156 μg/mL for the different compounds. Allicin showed the greatest efficacy (i.e., lowest MIC and MFC) against *Bsal* zoospores followed by curcumin, Pond Pimafix^®^, thymol, 6-gingerol, and Virkon^®^ Aquatic, respectively. Our results provide evidence that plant-derived fungicides are effective at inhibiting and killing *Bsal* zoospores in vitro and may be useful for in situ treatment. Additional studies are needed to estimate the efficacy of these fungicides at inactivating *Bsal* in the environment and treating *Bsal*-infected amphibians.

## 1. Introduction

Emerging fungal diseases are a major threat to plant, human, and animal health [1]. In wildlife, several fungal pathogens have recently emerged and are causing substantial losses to global biodiversity [1]. These pathogens are found across an array of ecosystems and taxa, including bats (white-nose syndrome caused by *Pseudogymnoascus destructans*) [2], soft corals (sea-fan aspergillosis caused by *Aspergillus sydowii*) [3], bees (colony collapse disorder caused by *Nosema* sp.) [4], snakes (snake fungal disease caused by *Ophidiomyces ophiodiicola*) [5], and amphibians (chytridiomycosis caused by *Batrachochytrium dendrobatidis* and *Batrachochytrium salamandrivorans*) [6,7]. Devising disease management strategies to combat emerging fungal pathogens in wildlife populations is an urgent global conservation priority [8,9].

The disease, amphibian chytridiomycosis, caused by *Batrachochytrium dendrobatidis* (*Bd*) and *B. salamandrivorans* (*Bsal*), is responsible for the greatest known loss of vertebrate biodiversity attributable to a pathogen [1]. Chytridiomycosis has contributed to the decline of an estimated 500 amphibian species [10]. The majority of these declines are attributed to *Bd* causing disease in anurans; however, *Bsal*, which was recently discovered and seems more pathogenic to salamanders, is quickly emerging [10]. *Bsal* is currently spreading across Europe and causing mass mortality events of several *Salamandridae* species [6,11]. Presumably originating from Asia, *Bsal* is thought to have spread to Europe via the wildlife trade [6,12]. *Bsal* has yet to be confirmed in North America, but may arrive soon, considering the United States (U.S.) and Canada comprise over half of the global amphibian imports [13,14]. In the U.S., especially the southeastern region, *Bsal* introduction is predicted to have severe negative impacts on endemic species [12,15].

Despite great advancements in understanding chytridiomycosis and its threats, successful in situ management to combat *Bd* and *Bsal* in infected areas has been limited [9,16]. Fungicides are an appealing management option due to their ability to treat the environment and infected individuals [16]. In a series of simulations comparing disease management strategies, Drawert et al. [17] reported that antifungal treatment was more effective than host density reduction during the epizootic phase of disease invasion. Further, due to the potential for frequency-dependent transmission of *Bsal* [18], treatment of the environment or animals might be more effective than population management strategies. Plant-derived fungicides are a type of antifungal treatment that may be less toxic to target amphibian species, non-target wildlife, and the environment compared to pesticides and antifungal medications. Despite this potential, only one study investigating the effects of plant-derived fungicides on chytrid fungi has been published. Silva et al. [19] tested three plant-derived fungicides (antifungal compounds of turmeric, garlic, and ginger) against *Bd* growth, and found all three were toxic to *Bd* zoospores, killing at least 50% of zoospores in 24 h. Although these results are promising, the environmental persistence of *Bsal* zoospores might be greater than *Bd* [20], and the life cycle of the two pathogens is different (e.g., *Bsal* has two zoospore forms) [7,20]. The objective of our study was to estimate the growth inhibition of five plant-derived fungicides and one common, commercially available chemical fungicide against *Bsal*, and identify potential candidates for future in situ treatment use.

## 2. Materials and Methods

### 2.1. Fungicides

We tested the following fungicides: thymol (thyme)**,** curcumin (turmeric)**,** allicin (garlic)**,** 6-gingerol (ginger), Pond Pimafix^®^ (2.5% West Indian bay tree; API), and Virkon^®^ Aquatic (Syndel, Ferndale, WA, USA; Table 1). All of the plant-derived fungicides (first five listed) are known to inhibit human fungal (i.e., *Candida* and *Aspergillus* spp.) and bacterial pathogens [21,22,23,24,25,26]. Thymol is commonly used in aquaculture as a nutritional supplement in fish feed, and has been found to improve fish health and increase immune response to various infections and diseases [27,28]. The fungicides, 6-gingerol (200 μg/mL), allicin (3.375 μg/mL), curcumin (6 μg/mL), and Virkon^®^ S (1%; Syndel, Ferndale, WA, USA), similar in formulation to Virkon^®^ Aquatic, are all toxic to *Bd* zoospores [19,29]. Virkon^®^ S was also tested against *Bsal* and can kill zoospores both at 0.5% (5-min exposure) and 1% (1-min exposure) concentrations [30]. We selected Virkon^®^ Aquatic because it is formulated based on the same active ingredients as Virkon^®^ S, however, it was specifically developed for aquatic applications. Pimafix^®^ (1% West Indian bay tree extract) has been used in experimental aquatic organisms (e.g., perch, eels, horseshoe crabs) to prevent or treat fungal and parasitic infections (i.e., *Gyrodactylus turnbuli* in guppies) [31,32,33,34]. We chose Pond Pimafix^®^ rather than Pimafix^®^ because it is slightly higher in West Indian bay tree concentration (2.5% rather than 1%) and is marketed towards application in a pond setting rather than aquaria. Based on their proven efficacy against human and other fungal pathogens, we hypothesized that these fungicides would be effective at inhibiting *Bsal*, although growth would be concentration dependent, and tested the concentrations in Table 1. Following standard practice in microbial research [35,36], we estimated the Minimum Inhibitory Concentration (MIC) and Minimum Fungicidal Concentration (MFC) as indices of concentration-dependent growth inhibition.

### 2.2. Bsal Culturing and Zoospore Harvesting

*Bsal* (isolate AMFP13/1) [37] was obtained from Frank Pasmans and An Martel (Ghent University), and was grown and maintained in half-strength TGhL broth (8 g tryptone, 2 g gelatin hydrolysate, 1 g lactose per liter of distilled water) [38] in 25 cm^2^ cell culture flasks at 14 °C. To maximize zoospore harvest, we pipetted 1 mL of active culture onto TGhL agar plates and incubated the plates for 5–7 days at 14 °C until apparent sporangia formation and zoospore release was observed. We carefully scraped the TGhL agar plates using a sterile cell scraper to remove sporangia and suspended the collected sporangia in half-strength TGhL broth for 24 h at 14 °C to synchronize zoospore release. After 24 h, we collected the zoospores by filtering the solution through a 20-μm filter to remove sporangia, and enumerated zoospores from two aliquots of the filtered solution (i.e., 1:10 dilution of filtered zoospores in half-strength TGhL broth) using a hemocytometer. We averaged the zoospore counts between both aliquots to estimate zoospore concentration and diluted the solution to a final concentration of 1 × 10^6^ zoospores/mL, which was used for all experiments.

### 2.3. Minimum Inhibitory Concentration (MIC) of Fungicides against Bsal

We performed a broth microdilution method similar to Martel et al. [39] and Silva et al. [19] in 48-well plates (non-treated, sterile, polystyrene, Falcon^®^) to determine the MIC for each fungicide. The MIC is the minimal drug concentration that inhibits fungal growth [40]. Allicin and Virkon^®^ Aquatic were solubilized in sterile water and Pond Pimafix^®^ was added directly into half-strength TGhL broth. The fungicides, 6-gingerol, thymol, and curcumin, were insoluble in half-strength TGhL broth or water, thus, were solubilized in 100% methanol. All concentrations of these methanol-solubilized fungicides used in the experiments were at or below 1% methanol, the highest percentage tested of the solvent that did not significantly affect *Bsal* zoospore growth on average over 9 independent plates (Figure S1). Working stock solutions for each fungicide were made freshly in half-strength TGhL broth and filter sterilized before every assay. Six concentrations of fungicide solution were prepared immediately prior to plate application.

In each well, we mixed 100 μL of *Bsal* zoospores (1 × 10^6^ zoospores/mL) and 100 μL of fungicide solution diluted to each target concentration. Each plate included a positive control (100 μL zoospores + 100 μL half-strength TGhL broth per well), negative control (100 μL heat-killed zoospores + 100 μL half-strength TGhL broth per well), and media control (200 μL half-strength TGhL broth per well) [19]. Each fungicide concentration and control were included in five wells per plate (i.e., 45 of the 48 wells were used per plate). We created heat-killed zoospores by exposing them to 90 °C for 20 min [41], and cooled to room temperature before plate application. All fungicide concentrations and controls were run in five replicates (i.e., five wells) on each plate. Each trial (i.e., three plates with six fungicide concentrations and three controls that were completed in one day) was repeated three times. Overall, nine plates with six fungicide concentrations and three controls over three days were completed per fungicide (i.e., 45 wells per fungicide concentration and each control in total for each fungicide; see Figure S2).

All plates were incubated at 14 °C for 72 h, which is the optimum temperature for *Bsal* growth [37]. After incubation, we performed the MTT cell viability assay, optimized for *Bsal* by Lindauer et al. [42], to estimate fungicide growth inhibition and identify the MIC. MTT (3-(4,5-dimethylthiazol-2-yl)-2,5-diphenyltetrazolium bromide) is a tetrazolium salt used in the colorimetric assay that changes from yellow to purple when reduced to formazan crystals by living cells, hence an effective method to estimate cell growth [43,44]. To perform the MTT assay, we added and mixed 40 μL of MTT into each well and covered the plate in foil to limit light penetration. We incubated each plate at 14 °C for two hours. After incubation, we added and slowly mixed 280 μL of 20% SDS/50% DMF solution into each well to solubilize the formazan crystals, and read the plate immediately on a spectrophotometer (BioTek^®^ Synergy HT, Winooski, VT, USA) at 570 nm, the most sensitive wavelength for this assay [42]. To estimate cell viability from absorbance readings, we used the following slightly modified equation, previously described by Silva et al. [19]: (A570 nm (fungicide sample) − A570 nm (mean negative control))/(A570 nm (mean positive control) − A570 nm (mean negative control)) × 100. This standard equation can lead to values less than 0 (i.e., less than the negative control) or greater than 100% (i.e., greater than the positive control). Values less than 0 or greater than 100% were included in analyses. Cell viability was estimated as a percentage of the positive control growth (i.e., live *Bsal* zoospores in TGhL broth) [19]. The MIC was defined as the lowest fungicide concentration cell viability (%), calculated from absorbance readings, that was not significantly different (*p* < 0.05) from the negative control (i.e., heat-killed zoospores) [19,45].

### 2.4. Minimum Fungicidal Concentration (MFC) of Fungicides against Bsal

The MFC was estimated as confirmatory evidence that the MIC, as identified by the MTT assay, inhibited growth of *Bsal* in culture. We evaluated the MIC and two higher concentrations by adding each fungicide to untreated TGhL agar plates (i.e., not inoculated with *Bsal*) and microscopically evaluating zoospore growth. All samples (three fungicide concentrations) and controls (positive, negative, and media) were prepared in a 48-well plate as described previously and incubated at 14 °C for 72 h. Following incubation, we pipetted 100 μL from each well onto a 6-well TGhL agar plate (3 mL of TGhL agar per well), with one inoculation per well to ensure replicate independence. On one of the 6-well plates, we added one replicate per fungicide concentration and the controls (positive, negative and media), totaling 15 agar plates per trial (i.e., five agar plates per one 48-well plate x three 48-well plates per trial) and 45 agar plates per fungicide overall (i.e., fifteen agar plates per trial x three trials completed on three separate days; see Figure S3). Once plated, we incubated the agar plates at 14 °C for one week and examined each well microscopically (Nikon Eclipse TS100, 20× magnification) to identify any fungal growth. The MFC was defined as the lowest tested fungicide concentration in which no visible growth was seen on any agar plate after one week.

### 2.5. Statistical Analyses

#### 2.5.1. MIC Estimation

For each fungicide, the goal of our statistical analysis was to identify the lowest fungicide concentration where cell viability was not significantly different from the negative control. The primary factor of interest was “treatment” (i.e., six levels of concentrations tested), but we had two additional blocking variables in our design: “plate date” (i.e., three separate days that the three trials were completed) and “plate order” (i.e., three groupings of the first, second, and third plates completed across all three trials). Inasmuch as the MTT assay is a multi-step, time sensitive assay, we accounted for possible differences among plates depending on their order of assay completion within a trial. Because we had independent replicates within blocks (i.e., wells), we analyzed our experiment as a generalized randomized block design [46]. We first identified whether there were two or three-way interactions among the treatment and blocking variables, indicating that the MIC might vary among different treatment, plate date, and plate order combinations. A three-way interaction was included because each plate was treated separately; thus, we were able to analyze differences in MIC among all nine plates. We analyzed the six following statistical models:

Treatment + Plate order + Plate date,
Treatment * Plate date,
Treatment * Plate order,
Treatment * Plate date + Plate order,
Treatment * Plate order + Plate date, and
Treatment * Plate date * Plate order.

Models with interactions also included associated main effects. We fit each model using a generalized least-squares model (GLS) with normal errors that allowed for heterogeneous variance across the six fungicide treatments [47]. We compared the models using Akaike’s Information Criterion (AIC), and selected the model with lowest AIC value as the best fit model [48]. All data analyses were performed using statistical software program R (version 3.6.2) and the nlme package (v3.1-148) [49].

The assumption of normality of residuals was tested with the visual inspection of diagnostic normal Q-Q plots for the model of best fit for each fungicide (see Figure S4). Based on these plots, we determined the residuals were symmetrical but exhibited heavier tails than a normal distribution. Given our residuals were not normally distributed, we conducted a bootstrap analysis to test that our assumptions of normality did not bias our estimated MIC for each fungicide. The bootstrap analysis was performed by generating 1000 re-sampled datasets for each fungicide, refitting the GLS model (Treatment + Plate order + Plate date), and extracting the bootstrapped sampling distributions of our model coefficients. We identified the lowest treatment concentration for each fungicide where the 95% confidence interval of the bootstrapped sampling distribution overlapped zero (i.e., the lowest concentration for which cell viability was not significantly different from the negative control; see Tables S1–S5). For each fungicide, we compared the estimated MIC from the best fit GLS model and bootstrap analysis and found that all of our MICs were consistent between both methods, thus supporting that non-normal error structure did not bias our results. For Pond Pimafix^®^, the concentration we identified as the MIC (31.25 μg/mL) was the first concentration that was significantly less than zero. As noted above, the standard equation we used can lead to values less than 0 (i.e., cell viability less than the negative control) which are still indicative of no *Bsal* growth.

If a two-way or three-way interaction was present in our best fit model, this indicated that the MIC could vary depending on the plate order and plate date. In this case, we fit GLS models separately for each of the three trials (if there was no significant “plate order” interaction; three models) or for each of the nine plates separately (if there was a significant interaction between treatment, plate order, and plate date; nine models). From models with significant interactions, we extracted the *p*-values associated with each pairwise treatment comparison and the negative control, resulting in 18 *p*-values for “plate date” (i.e., 6 per model × 3 models) and 54 *p*-values for “plate order” (i.e., 6 per model × 9 models). To correct for multiple post hoc comparisons, we used a False Discovery Rate (FDR) correction in program R [50]. Using the corrected *p*-values, we selected the lowest concentration that was not significantly different from the negative control for each plate or trial. The MIC was determined as the most commonly occurring lowest concentration among the nine plates or three trials.

For the fungicide allicin, the dataset was zero-inflated due to minimal growth of *Bsal* among all treatments. Therefore, we converted cell viability to zeroes (i.e., 0% viability) and ones (i.e., >0% viability) and analyzed using a binomial generalized linear model with bias reduction (brglm; v0.7.2) [51]. We removed the highest concentration, 5 μg/mL, from the analysis as, erroneously, this concentration exhibited higher cell viability than the five lower tested concentrations (2.5, 1.25, 0.625, 0.313, 0.156 μg/mL). We microscopically analyzed each well of every plate before performing the MTT assay, and the 5 μg/mL wells showed no signs of growth or viability. Hence, we concluded that the “apparent” increased cell viability measured by the MTT assay was due to allicin debris that could not be removed via filtration at this higher concentration, resulting in erroneously providing a signal representing cell viability. The same model selection and MIC analysis procedure as described above was completed for allicin using brglm models.

#### 2.5.2. MFC Estimation

No statistical analyses were performed to estimate the MFC. The MFC was identified as the lowest concentration where no visible growth was seen microscopically on any agar plate after one week of incubation.

## 3. Results

The best fit model for 4 of 6 fungicides (6-gingerol, curcumin, Pond Pimafix^®^, Virkon^®^ Aquatic) included the three-way interaction (Table 2); therefore, we identified the MIC based on the most commonly occurring concentration among the nine plates and three plate dates that was not significantly different from the negative control. Allicin and thymol did not include a two-way or three-way interaction (Table 2); therefore, the MIC was selected based on the best fit model (Treatment + Plate order + Plate date). All allicin concentrations showed either significant or marginally significant reduced *Bsal* growth compared to the negative control (Table S6). Regardless of the two or three-way interactions, the MIC that was identified for each fungicide was consistent among the nine plates and three plate dates (Tables S6–S19), except for the thymol, which had a slightly lower MIC (20 μg/mL) among the three plate dates than the nine plates (25 μg/mL). However, the MIC for thymol (25 μg/mL) was selected based on the best fit model (Treatment + Plate order + Plate date).

**Figure 1:**
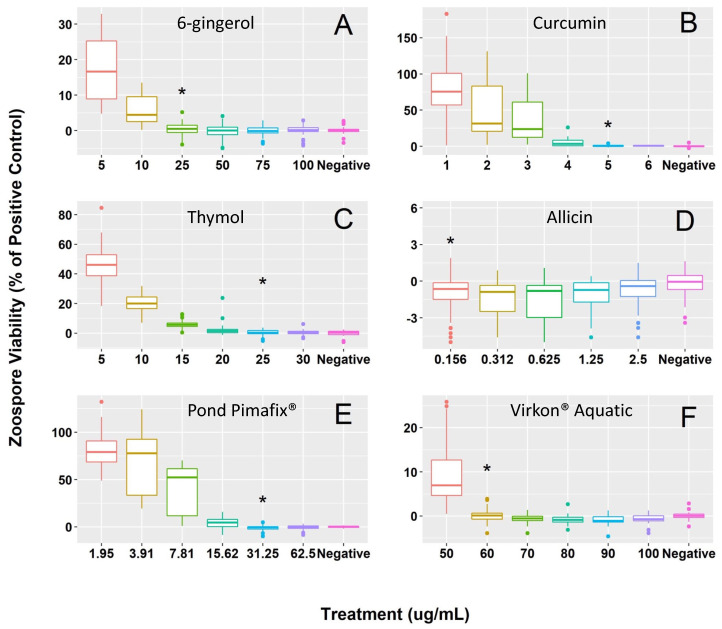
*Bsal* zoospore viability (% of the positive control) of the six tested concentrations and minimum inhibitory concentration [MIC] of (**A**) 6-gingerol [25 μg/mL], (**B**) curcumin [5 μg/mL], (**C**) thymol [25 μg/mL], (**D**) allicin [0.156 μg/mL], (**E**) Pond Pimafix^®^ [31.25 μg/mL], and (**F**) Virkon^®^ Aquatic [60 μg/mL]. An asterisk (*) is above the MIC for each fungicide. Each box plot represents *Bsal* zoospore viability relative to the positive control (*Bsal* zoospores in half-strength TGhL broth) for each concentration tested per fungicide (*n* = 45 wells per concentration across all plates). Midlines in each boxplot denote the median and the upper and lower sections of each box represent the first and third quartiles. Colored points extending beyond the boxplot represent outliers. As mentioned in the main text, “%” values can be negative based on the standard equation that we used if observed values are less than the negative control.

All fungicides exhibited inhibitory and fungicidal effects against *Bsal* growth, with estimated MIC concentrations ranging from 60 to 0.156 μg/mL across the six compounds (Figure 1). The MFC and MIC were identical for curcumin (5 μg/mL), allicin (0.156 μg/mL), Pond Pimafix^®^ (31.25 μg/mL), and Virkon^®^ Aquatic (60 μg/mL; Table 1). The MFC for thymol (35 μg/mL) and 6-gingerol (50 μg/mL) was higher than the MIC (25 μg/mL and 25 μg/mL respectively; Table 1). For killing *Bsal* zoospores, allicin showed the best efficacy (i.e., lowest MFC) followed by curcumin, Pond Pimafix^®^, thymol, 6-gingerol, and Virkon^®^ Aquatic, respectively (Figure 1).

## 4. Discussion

All of the fungicides we tested were successful at inhibiting growth and killing *Bsal* zoospores at relatively equivalent or lower concentrations compared to previous studies with other fungal pathogens. Silva et al. [19] tested the efficacy of 6-gingerol, allicin, and curcumin against *Bd* and found that all three were toxic to zoospores (i.e., killing at least 50% of the zoospore population within 24 h). However, the MICs of curcumin, 6-gingerol, and allicin for *Bd* zoospores were approximately 20%, 10× and 20× greater than for *Bsal*. Differences in efficacy could be partly related to fungicide exposure duration between our study (72 h) and Silva et al. [19] (24 h). *Bsal* zoospores also were more susceptible to plant-derived fungicides than several human pathogens tested in other studies. For example, the MICs for *Candida* spp. were approximately 2–50× greater than the MICs estimated for *Bsal* in this study (MICs = 6-gingerol 750–1000 μg/mL; thymol 39–78 μg/mL; allicin 8 μg/mL; curcumin 250–1000 μg/mL; West Indian bay tree 280 μg/mL) [21,52,53,54,55]. Similarly, except for West Indian bay tree (approx. 8× less susceptible), *Bsal* was approximately 6–50× more susceptible to plant-derived fungicides than *Aspergillus* spp. (MICs = 6-gingerol 750 μg/mL; thymol 125–200 μg/mL; allicin 8–32 μg/mL; curcumin 64 μg/mL, West Indian bay tree 3.9 μg/mL) [54,56,57,58,59]. Of the plant-derived fungicides we tested, allicin (0.156 μg/mL) and curcumin (5 μg/mL) had MICs and MFCs comparable to antifungal drugs previously tested on *Bsal* cultures (e.g., Voriconazole 0.125 μg/mL, 0.25 μg/mL; Itraconazole 0.006 μg/mL, 0.012 μg/mL; Terbinafine 0.2 μg/mL, 0.4 μg/mL) [41]. These results demonstrate that plant-derived fungicides can be highly effective at inhibiting *Bsal* growth. Overall, our results provide promise for treatment of *Bsal*-contaminated environments or infected animals with plant-derived fungicides.

These differences in MIC between *Bsal* and other fungi could be partly due to variation in morphological structures and growth thermal preferences. Specifically, for chytrid fungi, *Bsal* has a lower thermal tolerance (*Bsal* 10–15 °C; *Bd* 17–25 °C) [7,60] and several distinctive morphological differences compared to *Bd* (i.e., production of encysted zoospores, development of germ tubes from encysted zoospores, and increased presence of colonial thalli rather than monocentric thalli) [7,20,61]. It is possible that these fungicides are more effective at cooler temperatures or the life cycle and morphology of *Bsal* makes their zoospores more vulnerable. Furthermore, each of the fungicides possess different antimicrobial properties and utilize different mechanisms for inhibiting fungi and other pathogens. For example, allicin reacts with thiol groups and can inactivate essential enzymes [62], while curcumin has several modes of action, including inducing apoptosis pathways and increasing reactive oxygen species [63]. Thymol interferes with ergosterol biosynthesis, which increases membrane permeability and degrades cell function [55]. The mechanisms of 6-gingerol and West Indian bay tree are less well described but could include degradation and disruption of the cell membrane [64,65,66]. The specific mechanisms responsible for each fungicide inactivating *Bsal* are unknown and was outside of the scope of this study. Future research should evaluate the mechanisms by which each fungicide inactivates *Bsal* and assess whether combinations of fungicides could have synergistic effects. Combinatorial effects of allicin, curcumin, and 6-gingerol exhibited enhanced efficacy against *Bd* zoospores compared to single applications of each fungicide [19].

In aquaculture, plant-derived compounds have been used to boost immunity, reduce stress, prevent and treat infections and diseases (i.e., parasitic, bacterial, and fungal), and improve overall fish health [67,68,69,70,71]. Specifically, previous studies have proven the antimicrobial abilities and benefits of allicin, thymol, 6-gingerol, curcumin, and West Indian bay tree on aquatic organism health [27,33,72,73,74,75]. Despite their potential aquatic applications, plant-derived fungicides have yet to be extensively considered and investigated as a potential treatment for chytridiomycosis. Previous studies that tested use of chemical fungicides treating *Bd*-infected hosts and environments had varying levels of success [17,76,77], which demonstrates the challenges and sometimes inconsistencies between in vitro assays and in situ fungicide application. Despite these challenges, antifungal treatment remains a favorable in situ *Bsal* management strategy compared to other methods [17].

Plant-derived fungicides are a promising option for *Bsal* treatment; however, before environmental trials can occur, experiments testing the efficacy and toxicity of these fungicides in more natural conditions and against host animals are needed. Understanding the effects of plant-derived fungicides on the environment, target species, and non-target species is essential before broad-scale application should occur. Furthermore, environmental conditions (i.e., heat, humidity, temperature, oxygen) could affect the stability of plant-derived fungicides, thus their efficacy. Therefore, future experiments should investigate the efficacy of plant-derived fungicides against *Bsal* in pond water at varying temperatures and other abiotic conditions. These experiments also could assess the potential of using thermal refuges and plant-derived fungicides as a multi-pronged strategy for mitigating disease. Animal trials testing chronic and acute toxicity of plant-derived fungicides on both target amphibians and non-target species (i.e., vertebrates, invertebrates, and zooplankton) are also needed. Fungicide application could negatively impact the amphibian skin microbiome, which plays a critical role in host immunity [78]. Symbiotic bacterial species that aid in host protection against chytridiomycosis and other diseases could be altered, thus animal experiments should include measuring the effects of these fungicides on the skin microbiome. Selectivity indices could be established to understand the impacts of each fungicide on specific microbial species [79]. Future animal trials should include both *Bsal*-infected and uninfected individuals, because toxicity and negative microbial effects could vary depending on infection status and severity of disease. Future directions also should include measuring the efficacy of plant-derived fungicides against other amphibian pathogens. Plant-derived fungicides could serve as a comprehensive treatment option for hosts and the environment in disease systems where multiple pathogens could be present and co-infections occur. Estimating selectivity indices of each fungicide against different amphibian fungal pathogens (e.g., *Bd*, *Saprolegnia*) could be an approach to identify most effect treatments for co-infections [79].

Finally, we provide the first evidence that Virkon^®^ Aquatic is inhibitory and fungicidal to *Bsal* zoospores at 60 μg/mL (MIC and MFC), which constituted 0.06% of the final effective solution. The efficacy of Virkon^®^ S has been tested and resulted in the inactivation of *Bsal* zoospores in 2 min at 1% and 5 min at 2% effective solution [7]. Although the inhibitory concentration that we identified for Virkon^®^ Aquatic was considerably lower than Virkon^®^ S, it important to note that we incubated *Bsal* zoospores for 72 h compared to five minutes or less contact time in the Virkon^®^ S experiments. Thus, we recommend that shorter contact durations are evaluated for Virkon^®^ Aquatic before recommendations on its use as a disinfectant are made. It is also possible that Virkon^®^ Aquatic could be used to treat the environment. In an isolated aquatic system in Spain, *Bd* was eradicated by treating tadpoles with itraconazole and the environment with 1% Virkon^®^ S (Syndel, Ferndale, WA, USA) [80]. However, similar to plant-derived fungicides, the toxicity of Virkon^®^ Aquatic on amphibians and other aquatic organisms needs to be evaluated [81,82].

In conclusion, our results provide the first step to using plant-derived fungicides against *Bsal* and other emerging fungi and identify five candidate fungicides that warrant further study. Future research is still needed to determine their effects in aquatic systems under different environmental conditions, efficacy in clearing *Bsal* infection at varying disease states, toxicity in both hosts and non-target species, and potential use in treating other amphibian pathogens. The results of this study provide novel insight into the promising future of using plant-derived fungicides to manage fungal diseases in wildlife populations.

## Figures and Tables

**Table 1:** Concentrations tested and minimum inhibitory concentration (MIC) and minimum fungicidal concentration (MFC) estimated for evaluating the effect of six fungicides on *Bsal* zoospore growth.

Fungicide	Concentration Range	MIC	MFC
Thymol	30, 25, 20, 15, 10, 5 μg/mL	25 μg/mL	35 μg/mL
Curcumin	6, 5, 4, 3, 2, 1 μg/mL	5 μg/mL	5 μg/mL
6-gingerol	100, 75, 50, 25, 10, 5 μg/mL	25 μg/mL	50 μg/mL
Allicin	2.5, 1.25, 0.625, 0.313, 0.156 μg/mL	0.156 μg/mL	0.156 μg/mL
Pond Pimafix^®^	62.5, 31.25, 15.62, 7.81, 3.91, 1.95 μg/mL	31.25 μg/mL	31.25 μg/mL
Virkon^®^ Aquatic	100, 90, 80, 70, 60, 50 μg/mL	60 μg/mL	60 μg/mL

**Table 2:** Six generalized least-squares (GLS) models per fungicide testing for two-way or three-way interactions between treatment (i.e., six levels of concentrations tested), plate date (i.e., three separate days that the three trials were completed), and plate order (i.e., three groupings of the first, second, and third plates completed across all three trials).

Fungicide	Best Fit Model	ΔAIC ^1^	MIC ^2^
Thymol	**Treatment + Plate order + Plate date**	**0**	25 μg/mL No interaction model ^3^
	Treatment * Plate date	3.126	
	Treatment * Plate order	30.174	
	Treatment * Plate date + Plate order	2.867	
	Treatment * Plate order + Plate date	17.330	
	Treatment * Plate date * Plate order	51.655	
Curcumin	Treatment + Plate order + Plate date	286.771	5 μg/mL 5/9 plate order*date models ^3^ 2/3 plate date models
	Treatment * Plate date	26.616	
	Treatment * Plate order	305.615	
	Treatment * Plate date + Plate order	23.766	
	Treatment * Plate order + Plate date	294.345	
	Treatment * Plate date * Plate order	**0**	
6-gingerol	Treatment + Plate order + Plate date	277.191	25 μg/mL 6/9 plate order*date models 3/3 plate date models
	Treatment * Plate date	146.085	
	Treatment * Plate order	325.236	
	Treatment * Plate date + Plate order	116.422	
	Treatment * Plate order + Plate date	291.269	
	Treatment * Plate date * Plate order	**0**	
Allicin	**Treatment + Plate order + Plate date**	**0**	0.156 μg/mL No interaction model ^3^
	Treatment * Plate date	0.488	
	Treatment * Plate order	23.047	
	Treatment * Plate date + Plate order	3.271	
	Treatment * Plate order + Plate date	3.271	
	Treatment * Plate date * Plate order	3.271	
Pond Pimafix^®^	Treatment + Plate order + Plate date	358.097	31.25 μg/mL 5/9 plate order*date models 2/3 plate date models
	Treatment * Plate date	180.598	
	Treatment * Plate order	318.613	
	Treatment * Plate date + Plate order	167.370	
	Treatment * Plate order + Plate date	312.451	
	Treatment * Plate date * Plate order	**0**	
Virkon^®^ Aquatic	Treatment + Plate order + Plate date	78.395	60 μg/mL 7/9 plate order*date models 3/3 plate date models
	Treatment * Plate date	92.567	
	Treatment * Plate order	84.146	
	Treatment * Plate date + Plate order	80.262	
	Treatment * Plate order + Plate date	69.128	
	Treatment * Plate date * Plate order	**0**	

^1^ The models were compared using Akaike’s Information Criterion (AIC) and the model with the lowest AIC value was selected as the best fit model (bolded). The ΔAIC values (i.e., the difference between the model of best fit and each other model tested in the set) are displayed in the table. ^2^ The minimum inhibitory concentration (MIC) and the proportion of plate order by date (9 GLS models) and/or plate date models (3 GLS models) that selected the MIC (i.e., the most commonly occurring lowest concentration among the nine plates or three trials) using corrected *p*-values from each pairwise treatment comparison with the negative control. ^3^ For thymol and allicin, the MIC was inferred from the best fit model with only a main effect of Treatment (“No interaction model”). ^3^ Plate order by date models (plate order*date) were run for each of the nine plates separately if there was a significant interaction (*p* < 0.05) between treatment, plate order, and plate date.

## Data Availability

The data presented in this study are openly available in Zenodo at [10.5281/zenodo.7093361].

## Supplementary Materials

The following supporting information can be downloaded at: https://www.mdpi.com/article/10.3390/jof8101025/s1, Figure S1: *Bsal* zoospore viability (% of the positive control) of the six tested methanol concentrations and the highest concentration (HC) that did not significantly affect *Bsal* growth. Figure S2: A broth microdilution method was used to estimate the Minimum Inhibitory Concentration (MIC) for each fungicide. Figure S3: An agar plate growth method was used to estimate the Minimum Fungicidal Concentration (MFC) for each fungicide. Figure S4: Diagnostic normal Q-Q plots for the model of best fit for each fungicide, except allicin. Table S1: 95% confidence intervals for each thymol treatment concentration from the bootstrap analysis using 1000 re-sampled datasets to refit the GLS model (Treatment + Plate order + Plate date). Table S2: 95% confidence intervals for each curcumin treatment concentration from the bootstrap analysis using 1000 re-sampled datasets to refit the GLS model (Treatment + Plate order + Plate date). Table S3: 95% confidence intervals for each 6-gingerol treatment concentration from the bootstrap analysis using 1000 re-sampled datasets to refit the GLS model (Treatment + Plate order + Plate date). Table S4: 95% confidence intervals for each Pond Pimafix^®^ treatment concentration from the bootstrap analysis using 1000 re-sampled datasets to refit the GLS model (Treatment + Plate order + Plate date). Table S5: 95% confidence intervals for each Virkon^®^ Aquatic treatment concentration from the bootstrap analysis using 1000 re-sampled datasets to refit the GLS model (Treatment + Plate order + Plate date). Table S6: Binomial generalized linear model (brglm) with bias reduction of best fit model (Treatment + Plate order + Plate date) comparing *Bsal* zoospore viability (% of positive control) of five tested allicin concentrations to the negative control. Table S7: Generalized least square model (GLS) of best fit model (Treatment + Plate order + Plate date) comparing *Bsal* zoospore viability (% of positive control) of six tested thymol concentrations to the negative control. Table S8: Corrected *p*-values from each pairwise thymol treatment comparison (six levels of concentrations tested) with the negative control from generalized least-squares models fit for each of the nine plates separately showing the lowest concentration that was not significantly different from the negative control for each plate with bolded *p*-value. Table S9: Corrected *p*-values from each pairwise curcumin treatment comparison (six levels of concentrations tested) with the negative control from generalized least-squares models fit for each of the nine plates separately showing the lowest concentration that was not significantly different from the negative control for each plate with bolded *p*-value. Table S10: Corrected *p*-values from each pairwise 6-gingerol treatment comparison (six levels of concentrations tested) with the negative control from generalized least-squares models fit for each of the nine plates separately showing the lowest concentration that was not significantly different from the negative control for each plate with bolded *p*-value. Table S11: Corrected *p*-values from each pairwise allicin treatment comparison (five levels of concentrations tested) with the negative control from binomial generalized least-squares models with bias reduction fit for each of the nine plates separately showing the lowest concentration that was not significantly different from the negative control for each plate with bolded *p*-value. Table S12: Corrected *p*-values from each pairwise Pond Pimafix^®^ treatment comparison (six levels of concentrations tested) with the negative control from generalized least-squares models fit for each of the nine plates separately showing the lowest concentration that was not significantly different from the negative control for each plate with bolded *p*-value. Table S13: Corrected *p*-values from each pairwise Virkon^®^ Aquatic treatment comparison (six levels of concentrations tested) with the negative control from generalized least-squares models fit for each of the nine plates separately showing the lowest concentration that was not significantly different from the negative control for each plate with bolded *p*-value. Table S14: Corrected *p*-values from each pairwise thymol treatment comparison (six levels of concentrations tested) with the negative control from generalized least-squares models fit for each of the three plate dates separately showing the lowest concentration that was not significantly different from the negative control for each plate with bolded *p*-value. Table S15: Corrected *p*-values from each pairwise curcumin treatment comparison (six levels of concentrations tested) with the negative control from generalized least-squares models fit for each of the three plate dates separately showing the lowest concentration that was not significantly different from the negative control for each plate with bolded *p*-value. Table S16: Corrected *p*-values from each pairwise 6-gingerol treatment comparison (six levels of concentrations tested) with the negative control from generalized least-squares models fit for each of the three plate dates separately showing the lowest concentration that was not significantly different from the negative control for each plate with bolded *p*-value. Table S17: Corrected *p*-values from each pairwise allicin treatment comparison (five levels of concentrations tested) with the negative control from binomial generalized least-squares models with bias reduction fit for each of the three plate dates separately showing the lowest concentration that was not significantly different from the negative control for each plate with bolded *p*-value. Table S18: Corrected *p*-values from each pairwise Pond Pimafix^®^ treatment comparison (six levels of concentrations tested) with the negative control from generalized least-squares models fit for each of the three plate dates separately showing the lowest concentration that was not significantly different from the negative control for each plate with bolded *p*-value. Table S19: Corrected *p*-values from each pairwise Virkon^®^ Aquatic^®^ treatment comparison (six levels of concentrations tested) with the negative control from generalized least-squares models fit for each of the three plate dates separately showing the lowest concentration that was not significantly different from the negative control for each plate with bolded *p*-value. Minimum Inhibitory Concentration Estimation Process for Each Fungicide (Tables S6–S19). **Thymol (25 μg/mL; Tables S7, S8 and S14):** There was no two-way or three-way interaction between treatment, plate date, or plate order for thymol; therefore, the MIC was selected based on the best fit model (Treatment + Plate order + Plate date). The MIC was defined as the lowest treatment concentration that was not significantly different from the negative control. Based on the generalized least square model (GLS) of best fit model (Treatment + Plate order + Plate date), the MIC was determined to be 25 μg/mL (*p*-value = 0.454). Based on the GLS model fit for each of the nine plates, ~56% (5/9 plates) identified 25 μg/mL, ~33% (3/9 plates) identified 20 μg/mL, and ~11% (1/9 plates) identified 15 μg/mL as the MIC. Based on the GLS model fit for the three plate dates, ~67% (2/3 plates) identified 20 μg/mL and ~33% (1/3 plates) identified 25 μg/mL as the MIC. Overall, the selected MIC for the nine plates and three plates dates was 25 μg/mL, which was identical to the MIC determined by the best fit model (Treatment + Plate order + Plate date). **Curcumin (5 μg/mL; Tables S9 and S15):** There was a three-way interaction between treatment, plate date, and plate order for curcumin; therefore, the MIC was selected based on the best fit model (Treatment * Plate order * Plate date). We identified the MIC based on the most commonly occurring concentration among the nine plates and three plate dates that was not significantly different from the negative control. Based on the GLS model fit for each of the nine plates, ~11% (1/9 plates) identified 6 μg/mL, ~67% (5/9 plates) identified 5 μg/mL, and ~33% (3/9 plates) identified 4 μg/mL as the MIC. Based on the GLS model fit for the three plate dates, ~67% (2/3 plates) identified 5 μg/mL and ~33% (1/3 plates) identified 4 μg/mL as the MIC. Overall, the MIC for curcumin was determined to be 5 μg/mL. **6-gingerol (25 μg/mL; Tables S10 and S16):** There was a three-way interaction between treatment, plate date, and plate order for 6-gingerol; therefore, the MIC was selected based on the best fit model (Treatment * Plate order * Plate date). We identified the MIC based on the most commonly occurring concentration among the nine plates and three plate dates that was not significantly different from the negative control. Based on the GLS model fit for each of the nine plates, ~22% (2/9 plates) identified 50 μg/mL, ~67% (6/9 plates) identified 25 μg/mL, and ~11% (1/9 plates) identified 10 μg/mL as the MIC. Based on the GLS model fit for the three plate dates, 100% (3/3 plates) identified 25 μg/mL as the MIC. Overall, the MIC for 6-gingerol was determined to be 25 μg/mL. **Allicin (0.156 μg/mL; Tables S6, S11 and S17):** There was no two-way or three-way interaction between treatment, plate date, or plate order for allicin; therefore, the MIC was selected based on the best fit model (Treatment + Plate order + Plate date). The MIC was defined as the lowest treatment concentration that was not significantly different from the negative control. Based on the Binomial generalized linear model (brglm) with bias reduction of the best fit model (Treatment + Plate order + Plate date), the MIC was determined to be 0.156 μg/mL (*p*-value = 0.016). All allicin concentrations showed either significant or marginally significant reduced growth compared to the negative control, which indicates each successfully inhibited Bsal growth. Based on the brglm model fit for each of the nine plates, 100% (9/9 plates) identified 0.156 μg/mL as the MIC. Based on the brglm model fit for the three plate dates, 100% (3/3 plates) identified 0.156 μg/mL as the MIC. Overall, the selected MIC for the nine plates and three plates dates was 0.156 μg/mL, which was identical to the MIC determined by the best fit model (Treatment + Plate order + Plate date). **Pond Pimafix^®^ (31. 25 μg/mL; Tables S12 and S18):** There was a three-way interaction between treatment, plate date, and plate order for Pond Pimafix^®^; therefore, the MIC was selected based on the best fit model (Treatment * Plate order * Plate date). We identified the MIC based on the most commonly occurring concentration among the nine plates and three plate dates that was not significantly different from the negative control. Based on the GLS model fit for each of the nine plates, ~56% (5/9 plates) identified 31.25 μg/mL and ~44% (4/9 plates) identified 15.62 μg/mL as the MIC. Based on the GLS model fit for the three plate dates, ~67% (2/3 plates) identified 31.25 μg/mL and ~33% (1/3 plates) identified 15.62 μg/mL as the MIC. Overall, the MIC for curcumin was determined to be 31.25 μg/Ml. **Virkon^®^ Aquatic (60 μg/mL; Tables S13 and S19):** There was a three-way interaction between treatment, plate date, and plate order for Virkon^®^ Aquatic; therefore, the MIC was selected based on the best fit model (Treatment * Plate order * Plate date). We identified the MIC based on the most commonly occurring concentration among the nine plates and three plate dates that was not significantly different from the negative control. Based on the GLS model fit for each of the nine plates, ~11% (1/9 plates) identified 70 μg/mL, ~78% (7/9 plates) identified 60 μg/mL, and ~11% (1/9 plates) identified 50 μg/mL as the MIC. Based on the GLS model fit for the three plate dates, 100% (3/3 plates) identified 60 μg/mL as the MIC. Overall, the MIC for Virkon^®^ Aquatic was determined to be 60 μg/mL.
