# Supplementary material for: Efficacy of Plant-Derived Fungicides at Inhibiting Batrachochytrium salamandrivorans Growth
Source: J Fungi (Basel). 2022 Sep 28;8(10):1025. doi: 10.3390/jof8101025 (PMC9605044; doi:10.3390/jof8101025)
Supplement: Supplementary file 1 [file jof-08-01025-s001.zip › Figure S1_Methanol HC.pdf]

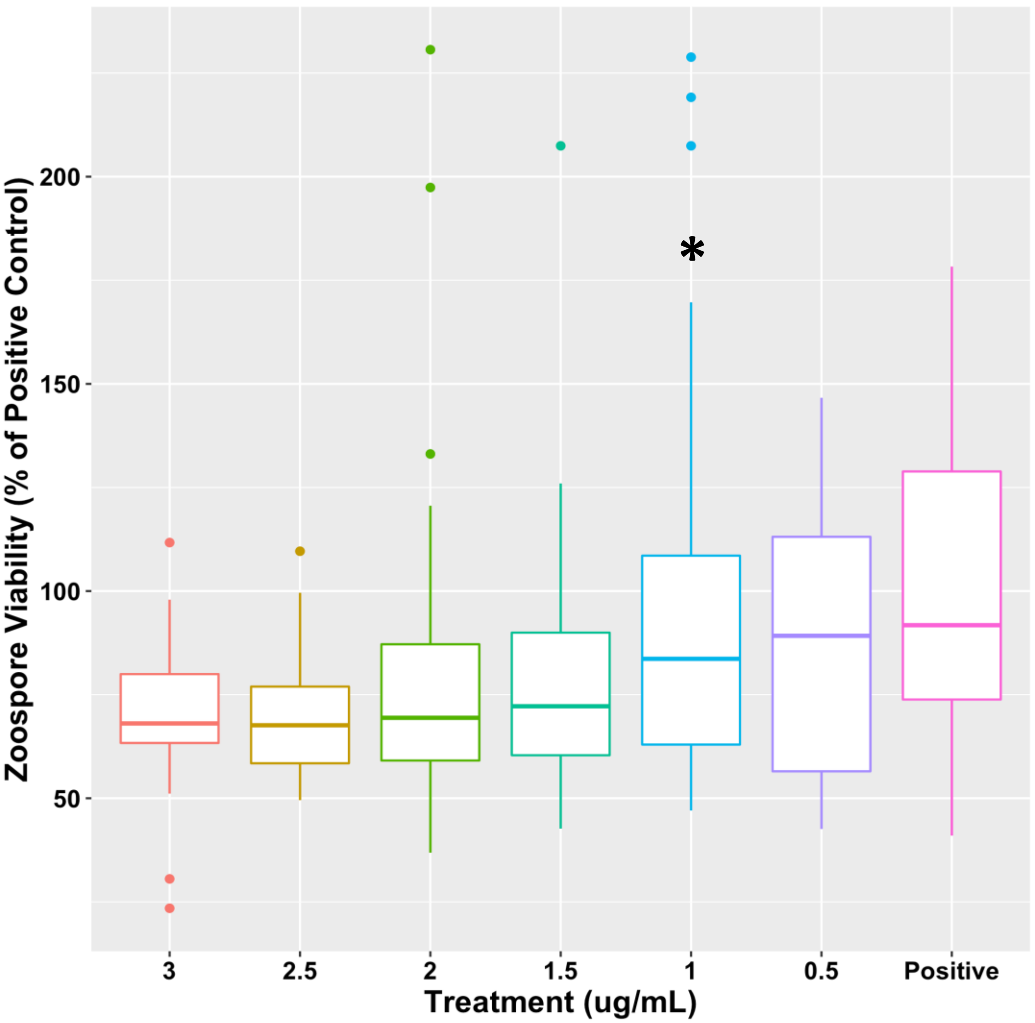

Figure S1. *Bsal* zoospore viability (% of the positive control) of the six tested methanol concentrations and the highest concentration (HC) that did not significantly affect *Bsal* growth (i.e., the highest concentration that was not significantly different than the positive control). An asterisk (*) is above the HC. The box plot represents *Bsal* zoospore viability relative to the positive control (*Bsal* zoospores in half-strength TGhL broth) for each concentration tested (n = 45 wells per concentration across all plates). Midlines in each boxplot denote the median and the upper and lower sections of each box represent the first and third quartiles. Colored points extending beyond the boxplot represent outliers.

To determine the HC for methanol, six generalized least-squares (GLS) models were tested for two-way or three-way interactions between treatment (i.e., six levels of concentrations tested), plate date (i.e., three separate days that the three trials were completed), and plate order (i.e., three groupings of the first, second, and third plates

completed across all three trials). From the best fit model (Treatment * Plate order * Plate date), the HC was 3% for the nine plates (7/9 plates) and 2% for the plate dates (2/3 plate dates). From the bootstrap analysis refitting the GLS model (Treatment + Plate order + Plate date), the HC was 1% (i.e., the highest treatment concentration where the 95% confidence interval of the bootstrapped sampling distribution overlapped zero). Based on these results, 1% was selected as the highest percentage tested of the methanol that did not significantly affect *Bsal* zoospore growth.
