# Supplementary material for: Efficacy of Plant-Derived Fungicides at Inhibiting Batrachochytrium salamandrivorans Growth
Source: J Fungi (Basel). 2022 Sep 28;8(10):1025. doi: 10.3390/jof8101025 (PMC9605044; doi:10.3390/jof8101025)
Supplement: Supplementary file 1 [file jof-08-01025-s001.zip › Figure S2_MIC Plate Setup.pdf]

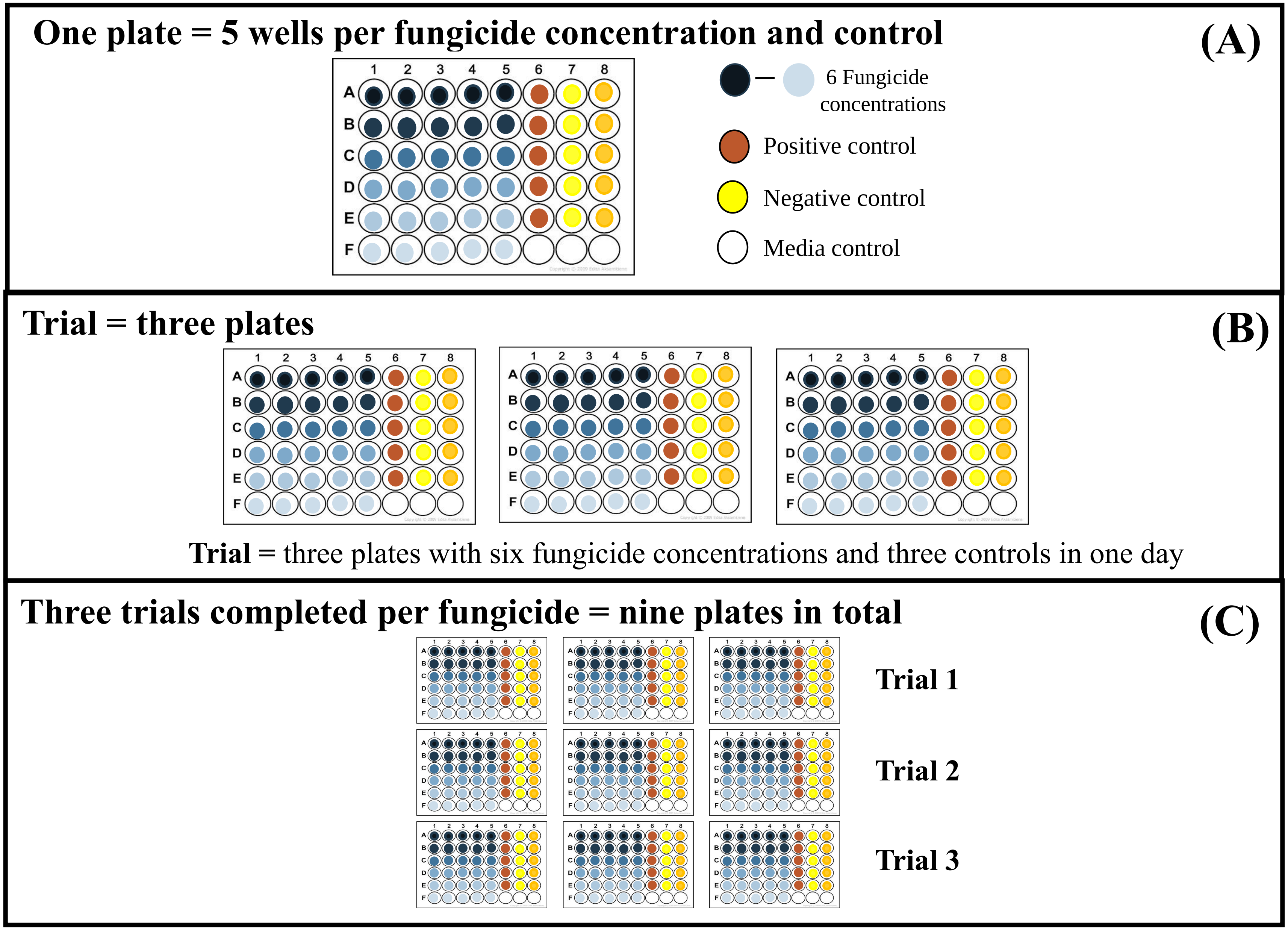
One plate = 5 wells per fungicide concentration and control
(A)
1 2 3 4 5 6 7 8
A B C D E F
6 Fungicide concentrations
Positive control
Negative control
Media control
Trial = three plates
(B)
Trial = three plates with six fungicide concentrations and three controls in one day
Three trials completed per fungicide = nine plates in total
(C)
Trial 1
Trial 2
Trial 3

Figure S2. A broth microdilution method was used to estimate the Minimum Inhibitory Concentration (MIC) for each fungicide**. (A)** On each 48-well plate (non-treated, sterile, polystyrene, Falcon®), six fungicide concentrations (100 μL of fungicide solution + 100 μL of $1x10^6$ *Bsal* zoospores per well), positive control (100 μL of $1x10^6$ *Bsal* zoospores + 100 μL half-strength TGhL broth per well), negative control (100 μL of $1x10^6$ heat-killed zoospores + 100 μL half-strength TGhL broth per well), and media control (200 μL half-strength TGhL broth per well) were added. Each fungicide concentration and control was included in five wells per plate (i.e., 45 of the 48 wells were used per plate). **(B)** Each trial (i.e., three plates with six fungicide concentrations and three controls that were completed in one day) was repeated three times. **(C)** Overall, three trials were completed for each fungicide. Specifically, nine plates with six fungicide concentrations and three controls over three days were completed per fungicide (i.e., 45 wells of each fungicide concentration and each control in total per fungicide).
