# Supplementary material for: Efficacy of Plant-Derived Fungicides at Inhibiting Batrachochytrium salamandrivorans Growth
Source: J Fungi (Basel). 2022 Sep 28;8(10):1025. doi: 10.3390/jof8101025 (PMC9605044; doi:10.3390/jof8101025)
Supplement: Supplementary file 1 [file jof-08-01025-s001.zip › Figure S3_MFC Plate Setup.pdf]

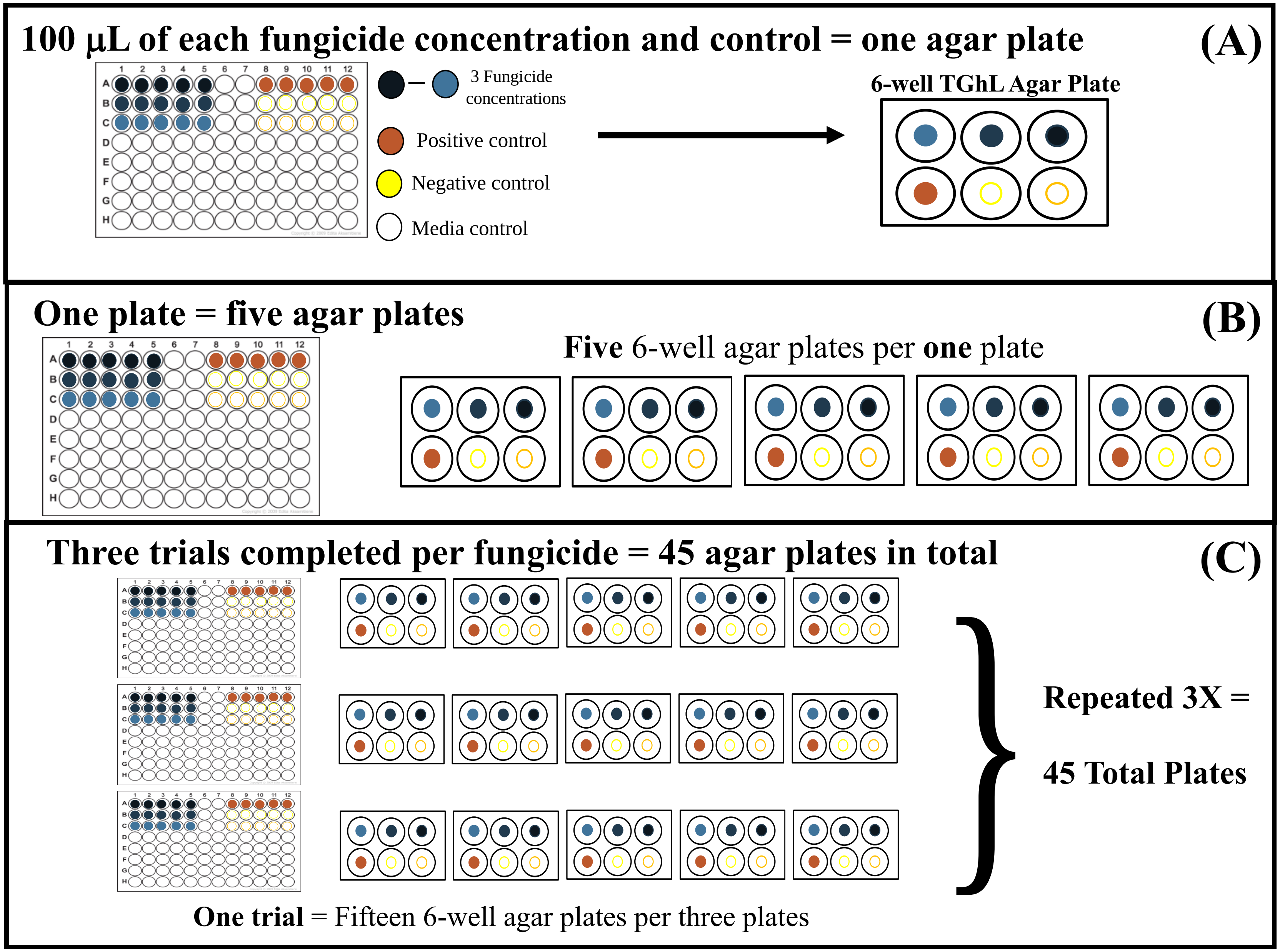
100 µL of each fungicide concentration and control = one agar plate
(A)
3 Fungicide concentrations
Positive control
Negative control
Media control
6-well TGhL Agar Plate
One plate = five agar plates
(B)
Five 6-well agar plates per one plate
Three trials completed per fungicide = 45 agar plates in total
(C)
Repeated 3X =
45 Total Plates
One trial = Fifteen 6-well agar plates per three plates

Figure S3. An agar plate growth method was used to estimate the Minimum Fungicidal Concentration (MFC) for each fungicide. **(A)** On each 48-well plate (non-treated, sterile, polystyrene, Falcon®), the estimated MIC and two higher fungicide concentrations (3 fungicide concentrations; 100 μL of fungicide solution + 100 μL of $1x10^6$ *Bsal* zoospores), positive control (100 μL of $1x10^6$ *Bsal* zoospores + 100 μL half-strength TGhL broth per well), negative control (100 μL of $1x10^6$ heat-killed zoospores + 100 μL half-strength TGhL broth per well), and media control (200 μL half-strength TGhL broth per well) were added. Each fungicide concentration and control were included in five wells per plate (i.e., 30 of the 48 wells were used per plate). After 72 hours of incubation at 14˚C, 100 μL from each well was pipetted onto a 6-well TGhL agar plate (3 mL of TGhL agar per well), with one inoculation per well to ensure replicate independence. **(B)** On one of the 6-well agar plates, we added one replicate per fungicide concentration and the controls (positive, negative and media). For each 48-well plate, there were five 6-well agar plates. **(C)** Each trial (i.e., three plates with three fungicide concentrations and three controls that were completed in one day) equaled fifteen 6-well agar plates per trial. Each trial was repeated three times, which equaled forty-five 6-well agar plates completed per fungicide.
