# Supplementary material for: Efficacy of Plant-Derived Fungicides at Inhibiting Batrachochytrium salamandrivorans Growth
Source: J Fungi (Basel). 2022 Sep 28;8(10):1025. doi: 10.3390/jof8101025 (PMC9605044; doi:10.3390/jof8101025)
Supplement: Supplementary file 1 [file jof-08-01025-s001.zip › Figure S4_Fungicide QQ Plots.pdf]

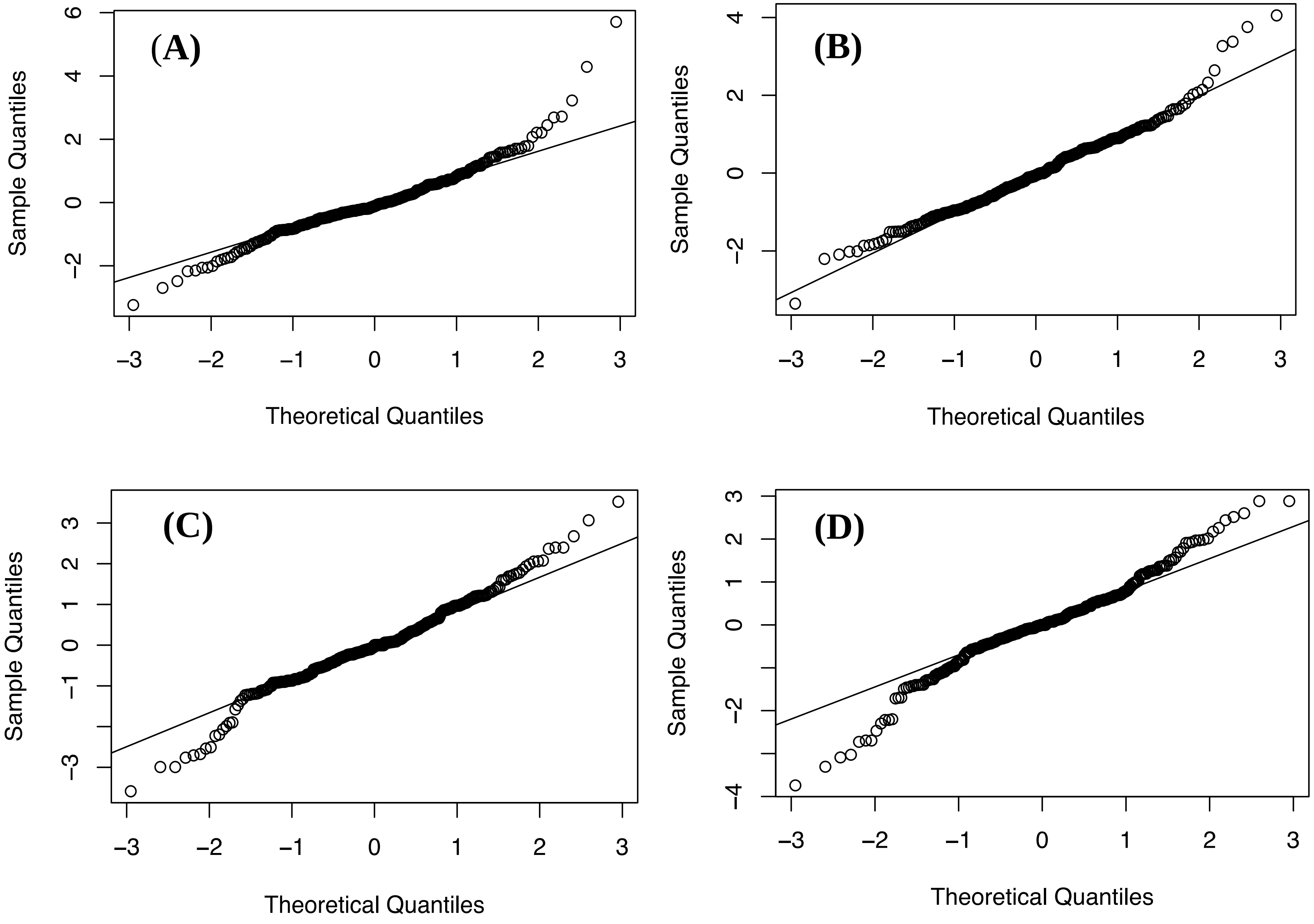
(A)
(B)
(C)
(D)
Sample Quantiles
Theoretical Quantiles
−3
−2
−1
0
1
2
3
4
6

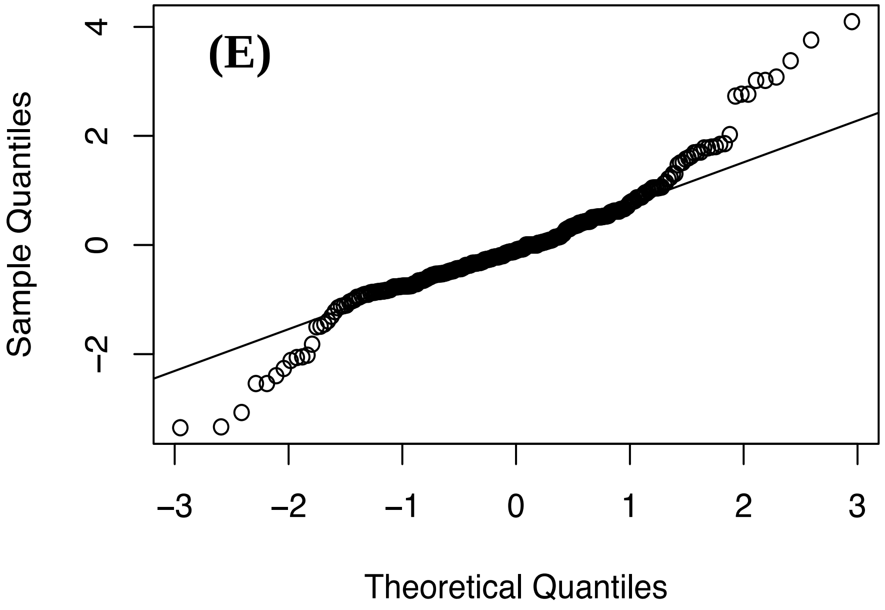

Figure S4. Diagnostic normal Q-Q plots for the model of best fit for each fungicide, including **(A)** thymol [Treatment + Plate order + Plate date], **(B)** curcumin [Treatment * Plate order * Plate date], **(C)** 6-gingerol [Treatment * Plate order * Plate date], **(D)** Pond Pimafix® [Treatment * Plate order * Plate date], and **(E)** Virkon® Aquatic [Treatment * Plate order * Plate date]. Allicin was analyzed using a binomial distribution, therefore, normality diagnostics were unnecessary, thus no normality diagnostics were performed.
