# Supplementary material for: Efficacy of Plant-Derived Fungicides at Inhibiting Batrachochytrium salamandrivorans Growth
Source: J Fungi (Basel). 2022 Sep 28;8(10):1025. doi: 10.3390/jof8101025 (PMC9605044; doi:10.3390/jof8101025)
Supplement: Supplementary file 1 [file jof-08-01025-s001.zip › Tables S1_S5_Fungicide Bootstrap 95 CI.pdf]

Table S1. 95% confidence intervals for each thymol treatment concentration from the bootstrap analysis using 1000 re-sampled datasets to refit the GLS model (Treatment + Plate order + Plate date).

	**Concentrations**	Intercept	5 ug/mL	10 ug/mL	15 ug/mL	20 ug/mL	25 ug/mL*	30 ug/mL
**95% CI**	2.5%	-0.284	42.091	18.425	4.980	1.082	-0.456	-0.254
	50%	0.475	45.389	20.159	5.880	2.130	0.273	0.402
	97.5%	1.192	49.252	22.052	6.810	3.523	1.102	1.160

*The MIC (denoted with an asterisks) was determined as the lowest treatment concentration where the 95% confidence interval overlapped zero.

Table S2. 95% confidence intervals for each curcumin treatment concentration from the bootstrap analysis using 1000 re-sampled datasets to refit the GLS model (Treatment + Plate order + Plate date).

	**Concentrations**	Intercept	1 ug/mL	2 ug/mL	3 ug/mL	4 ug/mL	5 ug/mL*	6 ug/mL
**95% CI**	2.5%	0.093	71.092	39.728	26.109	3.073	-0.136	0.040
	50%	0.590	81.563	49.489	33.313	4.428	0.277	0.467
	97.5%	1.148	93.879	60.171	41.149	5.950	0.696	0.879

*The MIC (denoted with an asterisks) was determined as the lowest treatment concentration where the 95% confidence interval overlapped zero.

Table S3. 95% confidence intervals for each 6-gingerol treatment concentration from the bootstrap analysis using 1000 re-sampled datasets to refit the GLS model (Treatment + Plate order + Plate date).

	**Concentrations**	Intercept	5 ug/mL	10 ug/mL	25 ug/mL*	50 ug/mL	75 ug/mL	100 ug/mL
**95% CI**	2.5%	-0.720	14.796	4.478	-0.163	-0.940	-0.762	-0.627
	50%	-0.162	17.530	5.668	0.439	-0.303	-0.196	-0.032
	97.5%	0.430	19.960	6.781	1.036	0.319	0.351	0.547

*The MIC (denoted with an asterisks) was determined as the lowest treatment concentration where the 95% confidence interval overlapped zero.

Table S4. 95% confidence intervals for each Pond Pimafix® treatment concentration from the bootstrap analysis using 1000 re-sampled datasets to refit the GLS model (Treatment + Plate order + Plate date).

	**Concentrations**	Intercept	1.95 ug/mL	3.91 ug/mL	7.81 ug/mL	15.62 ug/mL	31.25 ug/mL*	62.5 ug/mL
**95% CI**	2.5%	-1.284	75.261	58.234	33.106	2.216	-2.851	-2.285
	50%	-0.247	80.438	68.006	40.102	3.777	-1.786	-1.202
	97.5%	0.591	85.548	76.560	47.082	5.313	-0.728	-0.315

*The MIC (denoted with an asterisks) was determined as the lowest treatment concentration where the 95% confidence interval overlapped zero.

Table S5. 95% confidence intervals for each Virkon® Aquatic treatment concentration from the bootstrap analysis using 1000 re-sampled datasets to refit the GLS model (Treatment + Plate order + Plate date).

	**Concentrations**	Intercept	50 ug/mL	60 ug/mL*	70 ug/mL	80 ug/mL	90 ug/mL	100 ug/mL
**95% CI**	2.5%	-0.425	7.489	-0.323	-1.039	-1.209	-1.341	-1.023
	50%	-0.067	9.289	0.105	-0.623	-0.770	-0.928	-0.588
	97.5%	0.294	11.558	0.543	-0.258	-0.385	-0.485	-0.208

*The MIC (denoted with an asterisks) was determined as the lowest treatment concentration where the 95% confidence interval overlapped zero.
