# Supplementary material for: Efficacy of Plant-Derived Fungicides at Inhibiting Batrachochytrium salamandrivorans Growth
Source: J Fungi (Basel). 2022 Sep 28;8(10):1025. doi: 10.3390/jof8101025 (PMC9605044; doi:10.3390/jof8101025)
Supplement: Supplementary file 1 [file jof-08-01025-s001.zip › Tables S6_S19_Fungicide MIC Results.pdf]

Table S6. Binomial generalized linear model (brglm) with bias reduction of best fit model (Treatment + Plate order + Plate date) comparing *Bsal* zoospore viability (% of positive control) of five tested allicin concentrations to the negative control.

**Concentrations**	**Estimate**	**Stnd Error**	***Z* value**	***P*-value**†
Intercept	0.631	0.432	1.461	0.144
2.5 ug/mL	-0.797	0.461	-1.729	0.083
1.25 ug/mL	-1.291	0.4913	-2.670	0.009
0.625 ug/mL	-1.968	0.563	-3.498	< 0.001
0.312 ug/mL	-1.968	0.563	-3.498	< 0.001
0.156 ug/mL*	-1.156	0.481	-2.401	0.016

*The MIC (denoted with an asterisks) was defined as the lowest fungicide concentration cell viability (%), calculated from absorbance readings, that was not significantly different ($P < 0.05$) than the negative control (i.e., heat-killed zoospores). For allicin, the MIC was significantly different from the negative control because this concentration exhibited lower *Bsal* zoospore viability (% of positive control) than the negative control, indicated by a significant *P*-value (< 0.05). Overall, the MIC was determined to be 0.156 ug/mL, which was the lowest treatment concentration that exhibited lower *Bsal* zoospore viability (% of positive control) than the negative control.

†All allicin concentrations showed either significant or marginally significant reduced growth ($P < 0.05$) compared to the negative control, which indicates each successfully inhibited *Bsal* growth.

Table S7. Generalized least square model (GLS) of best fit model (Treatment + Plate order + Plate date) comparing *Bsal* zoospore viability (% of positive control) of six tested thymol concentrations to the negative control.

**Concentrations**	**Estimate**	**Stnd Error**	***T* value**	***P*-value**
Intercept	0.497	0.376	1.322	0.187
5 ug/mL	45.479	1.875	24.260	< 0.001
10 ug/mL	20.142	0.902	22.330	< 0.001
15 ug/mL	5.875	0.464	12.674	< 0.001
20 ug/mL	2.199	0.620	3.549	< 0.001
25 ug/mL*	0.284	0.378	0.750	0.454
30 ug/mL	0.426	0.356	1.195	0.233

*The MIC (denoted with an asterisks) was defined as the lowest fungicide concentration cell viability (%), calculated from absorbance readings, that was not significantly different ($P < 0.05$) than the negative control (i.e., heat-killed zoospores).

Table S8. Corrected *P*-values from each pairwise thymol treatment comparison (six levels of concentrations tested) with the negative control from generalized least-squares models fit for each of the nine plates separately showing the lowest concentration that was not significantly different from the negative control for each plate with bolded *P*-value.

Concentrations	Plate 1	Plate 2	Plate 3	Plate 4	Plate 5	Plate 6	Plate 7	Plate 8	Plate 9
Intercept	1.000	1.000	1.000	1.000	1.000	1.000	1.000	1.000	0.286
5 ug/mL	< 0.001	< 0.001	< 0.001	< 0.001	< 0.001	< 0.001	< 0.001	< 0.001	< 0.001
10 ug/mL	< 0.001	< 0.001	< 0.001	< 0.001	0.001	< 0.001	< 0.001	< 0.001	< 0.001
15 ug/mL	< 0.001	< 0.001	< 0.001	**0.052**	0.035	0.013	< 0.001	< 0.001	< 0.001
20 ug/mL	**0.334**	0.043	0.028	1.000	**0.989**	**0.995**	0.010	0.013	0.039
25 ug/mL*	0.430	**0.987**	**0.409**	0.989	0.404	0.987	**0.109**	**0.404**	**0.989**
30 ug/mL	0.234	0.875	0.409	0.903	0.989	0.989	0.672	0.409	0.444

* The MIC (denoted with an asterisks) was determined as the most commonly occurring lowest concentration among the nine plates.

Table S9. Corrected *P*-values from each pairwise curcumin treatment comparison (six levels of concentrations tested) with the negative control from generalized least-squares models fit for each of the nine plates separately showing the lowest concentration that was not significantly different from the negative control for each plate with bolded *P*-value.

Concentrations	Plate 1	Plate 2	Plate 3	Plate 4	Plate 5	Plate 6	Plate 7	Plate 8	Plate 9
Intercept	1.000	1.000	1.000	1.000	0.004	< 0.001	1.000	0.960	0.388
1 ug/mL	< 0.001	< 0.001	< 0.001	0.035	< 0.001	< 0.001	< 0.001	< 0.001	< 0.001
2 ug/mL	< 0.001	< 0.001	< 0.001	0.013	< 0.001	< 0.001	< 0.001	< 0.001	< 0.001
3 ug/mL	< 0.001	< 0.001	< 0.001	< 0.001	< 0.001	< 0.001	< 0.001	< 0.001	< 0.001
4 ug/mL	**0.053**	**0.251**	**0.613**	< 0.001	< 0.001	0.003	< 0.001	< 0.001	0.004
5 ug/mL*	0.960	0.386	0.956	**0.351**	0.001	**0.960**	**0.894**	**0.536**	**0.960**
6 ug/mL	0.002††	0.206	0.960	0.640	**0.059**	0.082	0.039††	0.125	0.769

*The MIC (denoted with an asterisks) was determined as the most commonly occurring lowest concentration among the nine plates.

†† *Bsal* zoospore viability (% of positive control) was slightly higher at 6 ug/mL compared to the MIC (5 ug/mL). Each well of every plate was microscopically analyzed before performing the MTT assay and these concentrations showed no signs of growth or viability; therefore, it was concluded that this increased cell viability was due to debris or assay application error.

Table S10. Corrected *P*-values from each pairwise 6-gingerol treatment comparison (six levels of concentrations tested) with the negative control from generalized least-squares models fit for each of the nine plates separately showing the lowest concentration that was not significantly different from the negative control for each plate with bolded *P*-value.

Concentrations	Plate 1	Plate 2	Plate 3	Plate 4	Plate 5	Plate 6	Plate 7	Plate 8	Plate 9
Intercept	1.000	1.000	1.000	1.000	1.000	1.000	1.000	1.000	1.000
5 ug/mL	< 0.001	< 0.001	< 0.001	< 0.001	< 0.001	< 0.001	< 0.001	< 0.001	< 0.001
10 ug/mL	< 0.001	< 0.001	< 0.001	< 0.001	< 0.001	**0.018**	< 0.001	0.002	0.023
25 ug/mL*	**0.958**	0.017	0.021	**0.861**	**0.515**	< 0.001[†]	**0.426**	**0.305**	**0.451**
50 ug/mL	0.122	**0.619**	**0.226**	0.976	0.594	< 0.001[†]	0.949	0.327	0.266
75 ug/mL	0.266	0.737	0.451	0.895	0.917	< 0.001[†]	0.949	0.396	0.451
100 ug/mL	0.244	0.426	0.294	0.451	0.949	< 0.001[†]	0.637	0.162	0.861

*The MIC (denoted with an asterisks) was determined as the most commonly occurring lowest concentration among the nine plates.

[†] Concentrations higher than the MIC occasionally exhibited lower *Bsal* zoospore viability (% of positive control) than the negative control, indicated by a significant *P*-value (< 0.05).

Table S11. Corrected *P*-values from each pairwise allicin treatment comparison (five levels of concentrations tested) with the negative control from binomial generalized least-squares models with bias reduction fit for each of the nine plates separately showing the lowest concentration that was not significantly different from the negative control for each plate with bolded *P*-value.

Concentrations	Plate 1	Plate 2	Plate 3	Plate 4	Plate 5	Plate 6	Plate 7	Plate 8	Plate 9
Intercept	0.892	0.892	0.892	0.892	0.8924	0.892	0.506	0.892	0.892
2.5 ug/mL	1.000	1.000	0.892	0.892	0.5521	1.000	1.000	0.506	0.506
1.25 ug/mL	1.000	0.552	0.892	0.552	0.5057	0.552	1.000	0.506	0.506
0.625 ug/mL	0.892	1.000	0.552	0.506	0.5521	0.552	1.000	0.506	0.506
0.312 ug/mL	0.892	1.000	0.552	0.506	0.5057	0.552	1.000	0.506	0.506
0.156 ug/mL*	**0.552**	**0.892**	**0.552**	**0.5521**	**0.552**	**0.552**	**1.000**	**0.506**	**0.506**

*The MIC (denoted with an asterisks) was determined as the most commonly occurring lowest concentration among the nine plates.

Table S12. Corrected *P*-values from each pairwise Pond Pimafix® treatment comparison (six levels of concentrations tested) with the negative control from generalized least-squares models fit for each of the nine plates separately showing the lowest concentration that was not significantly different from the negative control for each plate with bolded *P*-value.

Concentrations	Plate 1	Plate 2	Plate 3	Plate 4	Plate 5	Plate 6	Plate 7	Plate 8	Plate 9
Intercept	1.000	1.000	1.000	1.000	1.000	1.000	1.000	1.000	1.000
1.95 ug/mL	< 0.001	< 0.001	< 0.001	< 0.001	< 0.001	< 0.001	< 0.001	< 0.001	< 0.001
3.91 ug/mL	< 0.001	< 0.001	< 0.001	< 0.001	< 0.001	< 0.001	< 0.001	< 0.001	< 0.001
7.81 ug/mL	< 0.001	< 0.001	< 0.001	< 0.001	< 0.001	< 0.001	< 0.001	< 0.001	< 0.001
15.62 ug/mL	**0.217**	**< 0.001**[†††]	**0.410**	0.011	< 0.001	< 0.001	< 0.001	**0.738**	< 0.001
31.25 ug/mL*	0.410	< 0.001[†]	0.786	**0.631**	**0.410**	**0.410**	**0.233**	< 0.001[†]	**0.510**
62.5 ug/mL	0.463	< 0.001[†]	1.000	1.000	0.463	0.463	0.410	< 0.001[†]	0.463

*The MIC (denoted with an asterisks) was determined as the most commonly occurring lowest concentration among the nine plates.

[†] Concentrations higher than the MIC occasionally exhibited lower *Bsal* zoospore viability (% of positive control) than the negative control, indicated by a significant *P*-value (< 0.05).

[†††] For Plate 2, all concentrations had significant *P*-values (< 0.05). Concentrations 1.95, 3.91, and 7.81ug/mL exhibited higher *Bsal* zoospore viability (% of positive control) than the negative control, indicated by a significant *P*-values (< 0.05). Concentrations 15.62, 31.25, and 62.5 ug/mL exhibited lower *Bsal* zoospore viability (% of positive control) than the negative control, which was also indicated by a significant *P*-values (< 0.05). The MIC was determined to be 15.62 ug/mL, which was the lowest treatment concentration that exhibited lower *Bsal* zoospore viability (% of positive control) than the negative control.

Table S13. Corrected *P*-values from each pairwise Virkon® Aquatic treatment comparison (six levels of concentrations tested) with the negative control from generalized least-squares models fit for each of the nine plates separately showing the lowest concentration that was not significantly different from the negative control for each plate with bolded *P*-value.

Concentrations	Plate 1	Plate 2	Plate 3	Plate 4	Plate 5	Plate 6	Plate 7	Plate 8	Plate 9
Intercept	1.000	1.000	1.000	1.000	1.000	1.000	1.000	1.000	1.000
50 ug/mL	< 0.001	< 0.001	< 0.001	< 0.001	0.013	< 0.001	0.008	**0.106**	0.010
60 ug/mL*	**0.107**	**0.795**	**0.316**	**0.335**	**0.826**	0.009	**0.411**	0.444	**0.653**
70 ug/mL	0.633	0.072	0.044†	0.489	0.005†	**1.000**	0.455	0.332	0.616
80 ug/mL	0.653	0.779	0.001†	0.533	0.107	0.074	0.430	0.038†	0.455
90 ug/mL	0.004†	0.616	< 0.001†	0.413	0.001†	0.749	0.110	< 0.001†	0.779
100 ug/mL	0.966	0.010†	< 0.001†	0.447	0.008†	0.566	0.345	0.030†	0.485

*The MIC (denoted with an asterisks) was determined as the most commonly occurring lowest concentration among the nine plates.

† Concentrations higher than the MIC occasionally exhibited lower *Bsal* zoospore viability (% of positive control) than the negative control, indicated by a significant *P*-value (< 0.05).

Table S14. Corrected *P*-values from each pairwise thymol treatment comparison (six levels of concentrations tested) with the negative control from generalized least-squares models fit for each of the three plate dates separately showing the lowest concentration that was not significantly different from the negative control for each plate with bolded *P*-value.

Concentrations	Plate Date 1	Plate Date 2	Plate Date 3
Intercept	1.000	1.000	1.000
5 ug/mL	< 0.001	< 0.001	< 0.001
10 ug/mL	< 0.001	< 0.001	< 0.001
15 ug/mL	< 0.001	< 0.001	< 0.001
20 ug/mL*	**0.071**	**1.000**	< 0.001
25 ug/mL	0.447	0.907	**0.214**
30 ug/mL	0.385	1.000	0.204

*The MIC (denoted with an asterisks) was determined as the most commonly occurring lowest concentration among the three plate dates.

Table S15. Corrected *P*-values from each pairwise curcumin treatment comparison (six levels of concentrations tested) with the negative control from generalized least-squares models fit for each of the three plate dates separately showing the lowest concentration that was not significantly different from the negative control for each plate with bolded *P*-value.

Concentrations	Plate Date 1	Plate Date 2	Plate Date 3
Intercept	1.000	0.208	0.423
1 ug/mL	< 0.001	< 0.001	< 0.001
2 ug/mL	< 0.001	< 0.001	< 0.001
3 ug/mL	< 0.001	< 0.001	< 0.001
4 ug/mL	**0.081**	< 0.001	< 0.001
5 ug/mL*	0.823	**0.313**	**0.651**
6 ug/mL	0.041[††]	0.823	0.208

*The MIC (denoted with an asterisks) was determined as the most commonly occurring lowest concentration among the three plate dates.

[††] *Bsal* zoospore viability (% of positive control) was slightly higher at 6 ug/mL compared to the MIC (5 ug/mL) for Plate Date 1. Each well of every plate was microscopically analyzed before performing the MTT assay and this concentration showed no sign of growth or viability for any plates within this trial; therefore, it was concluded that this increased cell viability was due to debris or assay application error.

Table S16. Corrected *P*-values from each pairwise 6-gingerol treatment comparison (six levels of concentrations tested) with the negative control from generalized least-squares models fit for each of the three plate dates separately showing the lowest concentration that was not significantly different from the negative control for each plate with bolded *P*-value.

Concentrations	Plate Date 1	Plate Date 2	Plate Date 3
Intercept	1.000	1.000	1.000
5 ug/mL	< 0.001	< 0.001	< 0.001
10 ug/mL	< 0.001	< 0.001	< 0.001
25 ug/mL*	**0.070**	**0.070**	**0.070**
50 ug/mL	0.138	0.070	0.132
75 ug/mL	0.314	0.070	0.138
100 ug/mL	1.000	0.070	0.124

*The MIC (denoted with an asterisks) was determined as the most commonly occurring lowest concentration among the three plate dates.

Table S17. Corrected *P*-values from each pairwise allicin treatment comparison (five levels of concentrations tested) with the negative control from binomial generalized least-squares models with bias reduction fit for each of the three plate dates separately showing the lowest concentration that was not significantly different from the negative control for each plate with bolded *P*-value.

Concentrations	Plate Date 1	Plate Date 2	Plate Date 3
Intercept	0.809	0.809	0.608
2.5 ug/mL	0.809	0.456	0.314
1.25 ug/mL	0.809	0.141	0.157
0.625 ug/mL	0.456	0.141	0.157
0.312 ug/mL	0.456	0.141	0.171
0.156 ug/mL*	**0.456**	**0.157**	**0.608**

*The MIC (denoted with an asterisks) was determined as the most commonly occurring lowest concentration among the three plate dates.

Table S18. Corrected *P*-values from each pairwise Pond Pimafix® treatment comparison (six levels of concentrations tested) with the negative control from generalized least-squares models fit for each of the three plate dates separately showing the lowest concentration that was not significantly different from the negative control for each plate with bolded *P*-value.

Concentrations	Plate Date 1	Plate Date 2	Plate Date 3
Intercept	1.000	1.000	1.000
1.95 ug/mL	< 0.001	< 0.001	< 0.001
3.91 ug/mL	< 0.001	< 0.001	< 0.001
7.81 ug/mL	< 0.001	< 0.001	< 0.001
15.62 ug/mL	**0.319**	< 0.001	< 0.001
31.25 ug/mL*	0.008†	**0.189**	**0.304**
62.5 ug/mL	0.036†	0.758	0.184

*The MIC (denoted with an asterisks) was determined as the most commonly occurring lowest concentration among the three plate dates.
† Concentrations higher than the MIC occasionally exhibited lower *Bsal* zoospore viability (% of positive control) than the negative control, indicated by a significant *P*-values (< 0.05).

Table S19. Corrected *P*-values from each pairwise Virkon® Aquatic® treatment comparison (six levels of concentrations tested) with the negative control from generalized least-squares models fit for each of the three plate dates separately showing the lowest concentration that was not significantly different from the negative control for each plate with bolded *P*-value.

Concentrations	Plate Date 1	Plate Date 2	Plate Date 3
Intercept	1.000	1.000	1.000
50 ug/mL	< 0.001	< 0.001	< 0.001
60 ug/mL	**0.239**	**0.239**	**0.307**
70 ug/mL	0.008†	0.098	0.139
80 ug/mL	0.094	0.008†	0.063
90 ug/mL	0.001†	0.016†	0.034†
100 ug/mL	0.001†	0.234	0.234

*The MIC (denoted with an asterisks) was determined as the most commonly occurring lowest concentration among the three plate dates.

† Concentrations higher than the MIC occasionally exhibited lower *Bsal* zoospore viability (% of positive control) than the negative control, indicated by a significant *P*-values (< 0.05).
